# Habitat selection of a parasitoid mediated by volatiles informing on host and intraguild predator densities

**DOI:** 10.1007/s00442-015-3326-2

**Published:** 2015-05-07

**Authors:** Belén Cotes, Linda-Marie Rännbäck, Maria Björkman, Hans Ragnar Norli, Nicolai V. Meyling, Birgitta Rämert, Peter Anderson

**Affiliations:** Department of Plant Protection Biology, Integrated Plant Protection, Swedish University of Agricultural Sciences, P.O. Box 102, Växtskyddsvägen 3, Alnarp, SE-230 53 Sweden; Plant Health and Plant Protection Division, Norwegian Institute for Agricultural and Environmental Research (Bioforsk), Høgskoleveien 7, Ås, 1430 Norway; Department of Plant and Environmental Sciences, University of Copenhagen, Thorvaldsensvej 40, Frederiksberg C, 1871 Denmark; Department of Plant Protection Biology, Chemical Ecology, Swedish University of Agricultural Sciences, P.O. Box 102, Växtskyddsvägen 3, Alnarp, SE-230 53 Sweden

**Keywords:** *Delia radicum*, Entomopathogenic fungus, Foraging decision, Trade-off, *Trybliographa rapae*

## Abstract

To locate and evaluate host patches before oviposition, parasitoids of herbivorous insects utilize plant volatiles and host-derived cues, but also evaluate predator-derived infochemicals to reduce predation risks. When foraging in host habitats infested with entomopathogenic fungi that can infect both a parasitoid and its host, parasitoids may reduce the risk of intraguild predation (IGP) by avoiding such patches. In this study, we examined whether the presence of the entomopathogenic fungi *Metarhizium brunneum* and *Beauveria bassiana* in soil habitats of a root herbivore, *Delia radicum,* affects the behavior of *Trybliographa rapae*, a parasitoid of *D. radicum*. Olfactometer bioassays revealed that *T. rapae* avoided fungal infested host habitats and that this was dependent on fungal species and density. In particular, the parasitoid avoided habitats with high densities of the more virulent fungus, *M. brunneum*. In addition, host density was found to be important for the attraction of *T. rapae*. Volatiles collected from host habitats revealed different compound profiles depending on fungal presence and density, which could explain the behavior of *T. rapae*. We conclude that *T. rapae* females may use volatile compounds to locate high densities of prey, but also compounds related to fungal presence to reduce the risk of IGP towards themselves and their offspring.

**Electronic supplementary material:**

The online version of this article (doi:10.1007/s00442-015-3326-2) contains supplementary material, which is available to authorized users.

## Introduction

When two species which share a common prey resource interact with each other in terms of competition and predation per se this is known as intraguild predation (IGP) (Polis et al. 1989). The interaction can be either unidirectional or bidirectional and, in the latter case, the roles as IG predator and IG prey can shift. An example of a unidirectional IGP system is when a pathogen can infect both a herbivore and its parasitoid (Rosenheim et al. 1995; Straub et al. 2008).

Natural enemies of herbivores can utilize volatile secondary metabolites emitted by plants attacked by herbivores, i.e., herbivore-induced plant volatiles (HIPVs), when searching for prey (Dicke and Baldwin 2010). HIPVs and other host-derived cues have been shown to guide female parasitoids to locate and evaluate host patches before oviposition (Vet and Dicke 1992; Wäschke et al. 2013). It is also well established that host density plays a role when parasitoid females select suitable habitats for oviposition (Waage 1983; Connor and Cargain 1994; Hemachandra et al. 2007; Girling et al. 2011). The mechanism involved may be an increase in emitted HIPVs with higher host densities (Girling et al. 2011). However, parasitoid host foraging assumes trade-offs between oviposition sites (i.e., expected fitness gain) and the risk of predation (Weisser et al. 1994; Roitberg et al. 2010). In addition to host-derived cues, parasitoids may also respond to predator-derived infochemicals and avoid predation risks by diverting foraging to “safer” habitats (Raymond et al. 2000; Dicke and Grostal 2001; Nakashima et al. 2004; Meisner et al. 2011). Thus, by exhibiting variable anti-predator behavior, parasitoids can partly or fully avoid the potential disruptive impact of IGP (Snyder and Ives 2008).

Generalist entomopathogenic fungi can act as IG predators, as they infect insects at different trophic levels (Brooks 1993; Roy and Pell 2000; Furlong and Pell 2005). Entomopathogenic fungi are also known to affect the behavior of insects in different ways (Roy et al. 2006; Baverstock et al. 2010) and predators have been found to perceive and avoid fungal infested habitats, as well as infected prey and conspecifics (Meyling and Pell 2006; Ormond et al. 2011). Parasitoids may also be able to evaluate the IGP risk when foraging in fungal infested host habitats. Upon encountering fungal infected hosts in choice situations, both avoidance (Brobyn et al. 1988; Fransen and van Lenteren 1993) as well as a lack of avoidance (Lord 2001; Baverstock et al. 2005) have been demonstrated for different parasitoid species. During growth, fungi produce volatile metabolites (Crespo et al. 2008), which can be used as infochemicals by insects to avoid unsuitable habitats (Mburu et al. 2013). Furthermore, it is increasingly recognized that the host–habitat-selection behavior of natural enemies of herbivores can also be influenced by rhizosphere-inhabiting microbes via induced changes in the composition of released HIPVs (Guerrieri et al. 2004; Schausberger et al. 2012; Battaglia et al. 2013; Pineda et al. 2013; Soler et al. 2013).

In a host-pathogen-parasitoid system we studied the habitat choice of the wasp *Trybliographa rapae* Westwood (Hymenoptera: Figitidae), an oligophagous, solitary parasitoid of the below-ground larval stages of several *Delia* spp. (Diptera: Anthomyiidae) (Wishart and Monteith 1954). We used larvae of the cabbage root fly, *Delia radicum* L., a herbivore of roots of cruciferous plants in temperate climates (Finch 1989), feeding on white cabbage plants, as a model host habitat. Host habitat location by *T. rapae* females is mainly guided by olfactory cues with HIPVs released by the plant in combination with host-related cues (e.g., saliva, feces) informing the parasitoid of host presence (Brown and Anderson 1999; Neveu et al. 2002; Nilsson et al. 2012) and host density (Hemachandra et al. 2007). As pathogens, we used the generalist entomopathogenic fungi *Metarhizium brunneum* Petch and *Beauveria bassiana* (Balsamo) Vuillemin (Ascomycota: Hypocreales), which are ubiquitous in the soil environment (Meyling and Eilenberg 2007) and occur naturally at higher densities in plant rhizospheres than in the surrounding soil (Bruck 2010). These fungi can infect both *D. radicum* and *T. rapae* (Rännbäck et al. 2015), but it is currently unknown to what extent fungal presence in the host habitat influences foraging decisions by *T. rapae* females to reduce the risk of unidirectional IGP.

In the present study we addressed the following questions:

1. Do *T. rapae* females avoid *D. radicum* host habitats infested by different species of generalist entomopathogenic fungi?
2. If avoidance is observed, is this dependent on the densities of host larvae and fungus?
3. Is parasitoid selection in this system linked to changes in the composition of volatile emissions from the host habitats?

## Materials and methods

### Insect and plant material

The *D. radicum* culture used in the study originated from a laboratory culture at Warwick Crop Centre, Wellesbourne, UK, and was reared following the method described by Finch and Coaker (1969). The *T. rapae* culture originated from a laboratory culture from the University of Rennes, France, and the rearing method was modified from Neveu et al. (1996). In both insect cultures, we introduced field-collected insects from Sweden every year. The adult wasps and flies were kept in separate cabinets at 19 °C and a constant photoperiod of 16-h light:8-h dark (L:D). We provided the adult parasitoids with sucrose water and honey droplets and the adult flies with a mixture of honey, dried yeast, and milk powder, replacing the food twice a week. Female parasitoids used in the experiments were separated according to age (day of emergence), provided sucrose water and kept in rearing cabinets with males for mating.

We sowed seeds of white cabbage (*Brassica oleracea* var. *capitata* f. *alba* cv. Castello) in potting soil (Hasselfors Garden Special; Hasselfors Garden, Sweden) mixed with a slow-release fertilizer (Osmocote Pro 3–4 months, nitrogen–phosphorous–potassium 17–11–10 plus micronutrients; Weibulls Horto, Sweden) in 1.5- and 3.0-L plastic pots. The pots were then kept in a greenhouse chamber [22 ± 1 °C, 75 % relative humidity (RH), 16L:8D photoperiod] and the plants were used in experiments when they had eight and ten true leaves, corresponding to an age of 2 months.

Plant infestation involved placing 15 or 30 *D. radicum* eggs around the stem base of the plant, and covering them with damp soil, 9 days prior to the fungal inoculation. Since the egg hatching rate was not 100 % we established two levels of infestation: lightly infested plants (LIP; from one to ten larvae) or heavily infested plants (HIP; 15–30 larvae). Plants with 11–14 larvae were excluded from the analysis and uninfested plants (UP) used as controls. At the end of each experiment, we recorded the number of larvae on each plant.

### Fungal inoculation of plants

Two fungal isolates of *M. brunneum* (isolate KVL 04-57) and *B. bassiana* (isolate KVL 03-90), stored at −80 °C at the University of Copenhagen, Department of Plant and Environmental Sciences, were used in this study. For the preparation of cultures and conidial suspensions, we followed the methods described by Rännbäck et al. (2015). Concentrations of undiluted stock suspensions were established by serial dilutions and enumerations in a hemocytometer (0.0625 mm^2^, depth 0.200 mm; Fuchs–Rosenthal, VWR, Sweden). To determine conidia viability, we assessed the proportion of germinated conidia under 400× magnification after 24 h growth on Sabouraud dextrose agar. Germination was always more than 95 %. All stock suspensions were used on the day after preparation, at which time the fungal suspensions for the experiments were prepared.

We performed fungal inoculation of the plants 48 h prior to the experiments by manually pouring the fungal suspension around the stem base, in close proximity to the actively feeding *D. radicum* larvae. The HIP in 3.0- or 1.5-L pots received 40 or 20 mL of fungal suspension, respectively. To flush the conidia further down the root system, 20 or 10 mL, respectively, of tap water was added around the stem base immediately after the application of the fungal suspension. The same volumes of tap water were used for UP pots. We established two different densities of *B. bassiana* (*B. bassiana*–High, 1 × 10^9^ conidia mL^−1^; *B. bassiana*–Low, 1 × 10^8^ conidia mL^−1^) and *M. brunneum* (*M. brunneum*–High, 1 × 10^8^ conidia mL^−1^; *M. brunneum*–Low, 5 × 10^7^ conidia mL^−1^). These densities were based on a pilot study, in which the low densities resulted in <20 % larval mortality and the high densities resulted in >50 % larval mortality in *D. radicum*, as reported previously by Chandler and Davidson (2005) for *Metarhizium anisopliae*. As a lower inoculum level was required for *M. brunneum* compared with *B. bassiana* to achieve this effect, we assumed that the different fungal densities resulted in comparable infection levels of the two fungal species in *D. radicum* larvae.

### Olfactometer bioassay

**Fig. 1:**
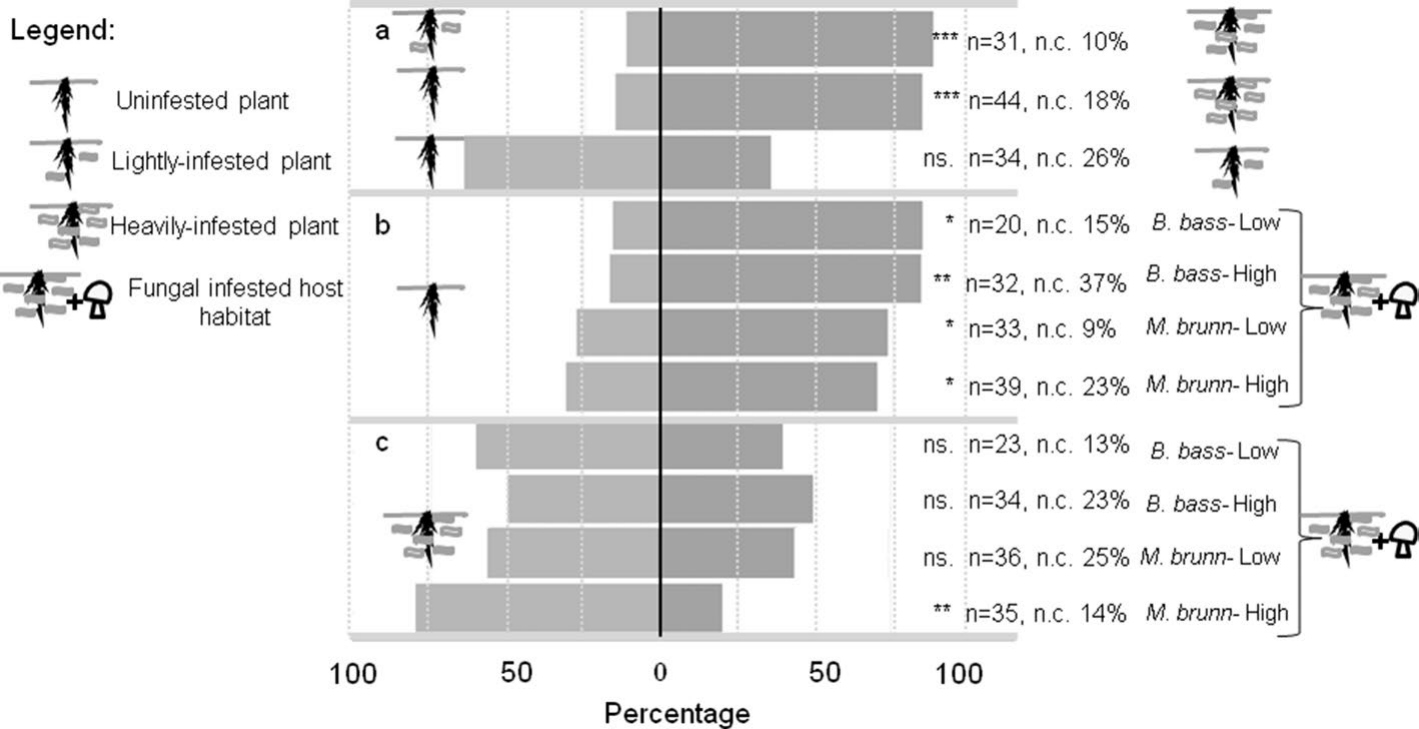
Behavioral response of *Trybliographa rapae* females offered **a** different infestation densities of *Delia radicum* larvae, **b** uninfested plants (UP) and heavily-infested plants (HIP) with low or high density of *Beauveria bassiana* or *Metarhizium brunneum*, and **c** HIP and HIP with low or high density of *B. bassiana* or *M. brunneum.* Test on two-tailed binomial distribution, *P < 0.05, **P < 0.01, ***P < 0.001, *ns* not significant, *n* numbers of female responders, *n*.*c*. percentages of female responders not making a choice (excluded from the statistical analysis)

We evaluated the responses of host-naïve *T. rapae* females to host density and fungal infested habitats in an olfactometer set-up as described by Jönsson et al. (2005). For host density responses, we tested three treatment combinations: UP vs. LIP, UP vs. HIP, and LIP vs. HIP (Fig. 1a). For responses to fungal infested habitats, the tested combinations were UP vs. HIP with *B. bassiana*–High, *B. bassiana*–Low, *M. brunneum*–High and *M. brunneum*–Low (Fig. 1b) and HIP vs. the same four fungal treatments (Fig. 1c).

All experiments were conducted in a climate chamber at 24 °C, 60 % RH and a light intensity of 1300 lux (measured at the Y-tube intersection), between 8 a.m. and 5 p.m. The air introduced into the set-up was pumped through a bottle (250 mL) with activated charcoal to eliminate surrounding odors, and then through a bottle with water to provide humidity (7-Ah, 12-V Micropump; KNF Neuberger, Germany). We adjusted the air flow rate to each arm of the olfactometer to 0.8 ± 0.2 L min^−1^ by a flow meter and then allowed the parasitoids to acclimatize to room conditions for at least 30 min before being tested in the olfactometer. A piece of black plastic tube (2 cm) covered the arm 1 cm from the junction of the Y-shaped glass tube [arm length 220 mm, 15 mm inner diameter (ID)], in order to calm the insect before it chose a direction. We tested parasitoids individually and recorded a choice when they moved 5 cm into one of the arms and stayed there for no less than 30 s. Parasitoids which did not make a choice within 5 min were discarded and excluded from the statistical analysis. After tests on five individuals we shifted the position of the stimuli in the olfactometer, in order to eliminate potential positional bias. Moreover, after ten individuals had been tested we renewed the Y-tube stimuli source. Thus, each replication, which consisted of ten females, lasted <2 h. After use, all corks, Teflon tubes and Y-tubes were rinsed with ethanol (70 %) and sterile water and the Y-tube was heated in an oven at 400 °C for 8 h between experimental days. We prepared the odor stimuli by carefully removing the plants (including soil) from the pot and placing them in a polyacetate cooking bag (35 × 43 cm) (Toppits; Melitta), attached tightly with tape to the olfactometer. Plants grown in different-sized pots (1.5 and 3 L) were tested in order to check whether stress in root system growth could influence parasitoid choice.

### Volatile collection

To collect volatiles, we carefully removed an entire plant (including soil) from the pot, placed it in the same type of cooking bag used in the olfactometer experiments and sealed it to the Teflon tubes (ThermoFisher Scientific, Santa Clara, CA) of the collection set-up with tape. Volatile collection involved the following seven treatments (only 3-L pots): empty bags, soil, UP, HIP, and HIP with *B. bassiana*–High, *M. brunneum*–High, and *M. brunneum*–Low, with five replicate plants for HIP and eight for the other treatments.

The volatile collection system comprised a tandem Teflon filter holder, so that the sampled odors passed through each adsorbent filter in sequence. Each Teflon tube was 25 mm long with 3 mm ID and the front filter contained 15 mm of Tenax GR (80–60 mesh) (Scantec; Lab AB, Partille, Sweden) and the second filter 15 mm Porapak-Q (80–100 mesh) (Scantec; Lab AB). Each filter tube was held by two stoppers of Teflon tube (2 mm ID) and glass wool. We inserted the filters into the polyacetate bags, placed a charcoal filter in the opposite corner to purify incoming air and drew out volatiles using a reversed aquarium Rena 300 pump (Mars Fishcare, Chalfont, PA) at a flow rate through the filters of 100 mL air min^−1^ before starting the experiment, a value measured and adjusted by a flow meter. Volatile collection continued for 4 h, under the same climate chamber conditions as in the olfactometer experiments.

For analysis, we eluted each pair of adsorbent filters in individual GC vials with 500 µL hexane (purity ≥99 %; Fluka, Buchs, Switzerland). Each GC vial received 2 µL of an internal standard mixture containing 250 ng µL^−1^ heptyl acetate and 250 ng µL^−1^ undecyl acetate and then all vials were capped and stored at −20 °C until analysis. Before gas chromatography–mass spectrometry (GC–MS) analysis, the samples were concentrated with a stream of nitrogen to approximately 30–40 µL.

### Chemical analysis and compound identification

The system used for sample analysis comprised an Agilent 6890 N GC connected to an Agilent 5973 MS and a deconvolution reporting system (version A.02.00; Agilent Technologies) for compound identification. The method for compound analysis and identification is identical to that described in detail by Thöming et al. (2014). The identity of six selected compounds [dimethyl disulfide, β-myrcene, *o*-xylene, 3-hexanol, (*Z*)-3-hexen-1-yl-acetate and 1,3-di-tert-butylbenzene] was verified by comparing mass spectra, Kovats index, and retention time with those obtained for synthetic standards (Sigma-Aldrich, Zwijndrecht, the Netherlands) on the same column. Relative amounts of identified compounds were calculated by dividing the peak area (using the area from the total ion chromatogram) by the area of the internal standard heptyl acetate. Only compounds found exclusively in treatments with *D. radicum*-infested plants were analyzed further, i.e., we excluded all compounds found in control samples (empty bags and soil) and UP.

### Statistical analysis

Data analysis began with data exploration, following the protocol described by Zuur et al. (2010) and using R software (R Core Team 2012). The first analysis examined the female responses of every single treatment combination (Fig. 1) to determine whether there was a preference for one of the two odor sources. A two-tailed binomial test was applied with the null hypothesis that the distribution of the parasitoids over the two arms of the olfactometer was 50:50.

The second analysis focused on factors that might have affected the response of parasitoids among all the different treatment combinations which included fungi (Fig. 1b, c). We modeled the probability of choosing a fungal infested host habitat (*π*_*i*_) as a function of several covariates by fitting a Bernoulli generalized linear model (GLM) with logistic link function log(*πi*) = *η*_*i*_. We assume that the* N*_*i*_ female parasitoids are independent, because each parasitoid in group *i* has the probability *π*_*i*_. The covariates used in the predictor function (*η*_*i*_) were two continuous variables (searching time and difference in larval density) and three factors (fungal treatment, arm orientation and pot size) as main terms (Table 1). Thus, the predictor function was written as follows:

$$\eta_{i} = {\text{Intercept}} + {\text{Search Time}}_{i} + {\text{Larvae Dif}}_{i} + {\text{Fungal Treatment}}_{i} + {\text{Arm Orientation}}_{i} + {\text{Potsize}}_{i}$$

and this model was built in a Bayesian framework using JAGS software (Plummer 2008), and implemented in the R2jags package (Su and Yajima 2012). A Bayesian approach has been suggested to describe how parasitoids forage in a patchy environment (Pierre and Green 2008).

**Table 1:** Covariates included in the Bernoulli generalized linear model for *Trybliographa rapae* behavioral choices

Response variable	Abbreviation in predictor function	Type of response variable	Description
Difference in larval density	Larvae dif_*i*_	Continuous	Number of *Delia radicum* larvae in heavily infested plants (HIP) with fungus minus number of larvae in non-infested plants [uninfested plants (UP) or HIP]
Searching time	Search time_*i*_	Continuous	Time (min) spent in one arm after crossing the border of 5 cm
Fungal treatments	Fungal treatment_*i*_	Factor (four levels)	*Beauveria bassiana*–High, *B. bassiana*-Low, *Metarhizium brunneum*–High, *M. brunneum*–Low
Arm orientation	Arm orientation_*i*_	Factor (two levels)	Right and left (arm orientation)
Pot size	Pot size_*i*_	Factor (two levels)	1.5- and 3.0-L pots

We ran our model with 50,000 iterations and three chains in the Markov chain Monte Carlo (MCMC) process and dropped the first 40,000 iterations as the burn-in period. A thinning rate of ten based on initial checks for convergence, resulted in 3000 observations for calculating the posterior distributions. The forward selection procedure was used as model selection and the deviance information criterion (DIC) (Spiegelhalter et al. 2002) was used to choose the model best explaining the factors influencing habitat choice by *T. rapae* females. Models with smaller DICs are preferable. Finally, we estimated the posterior parameter distributions and observed covariate data to predict the mean response for the fitted model and obtain 99 % confidence intervals for the fit.

Finally, we applied a principal component analysis (PCA) ordination technique to the relative amounts of identified compounds to graphically represent multivariate patterns in volatile emissions for plant treatments with or without fungi for different fungal species and fungal densities. Before the analysis was carried out, we normalized the variables and removed highly correlated compounds using variance inflation factors to assess which compounds were collinear (Montgomery and Peck 1992).

The PCA was based on the Euclidian distance between observations (scaling two) and the results displayed graphically in a PCA distance biplot using the vegan package (Oksanen et al. 2013). To assess differences in the volatiles between treatments, we used permutational multivariate ANOVA (PerMANOVA), which employs a permutation procedure to assess significance and thus does not rely on the assumption of multivariate normality (Anderson 2001).

We tested the effect of the treatments using ANOVA on peak areas for each compound using the Kruskal–Wallis non-parametric test and Bonferroni-corrected Mann–Whitney *U*-tests for post hoc comparisons.

## Results

### Choice of host habitat

The responses of *T. rapae* to volatiles emitted from *D. radicum*-infested habitats depended on the host densities of the plant pairs. Females were significantly attracted to volatiles from HIP compared with UP and LIP. However, the responses of the parasitoids were not significantly different when they were offered UP and LIP (Fig. 1a). When *T. rapae* encountered UP versus HIP with any fungus, females significantly preferred the plants with host odors, despite the presence of the fungus. This response was independent of fungal species or fungal density (Fig. 1b). On choosing between HIP and HIP with fungus added, females showed a significant avoidance response only for *M. brunneum*–High. Neither *B. bassiana* at any density nor *M. brunneum*–Low affected the choice by *T. rapae* between HIP and HIP with fungus (Fig. 1c).

### Factors affecting choice of host habitat with fungal presence

In the Bernoulli GLM we evaluated whether the distribution of choices of *T. rapae* among all the different treatment combinations was significantly affected by the factors listed in Table 1, i.e., which covariates influenced parasitoid responses. The response values were: avoidance (values 0) or attraction (values 1) towards a HIP with fungus.

We compared the first ten potential models including only main terms based on their DIC values (Online Resource 1). Model 9 had the smallest DIC, but model 7 had only a slightly higher DIC. Due to the small difference between these two values of DIC, we selected the simpler model 7 for model interpretation. Furthermore, model 7, including differences in larval density and fungal treatment, showed a good mixing of chains with the three lines of chains hovering around equilibrium (Online Resource 2).

**Fig. 2:**
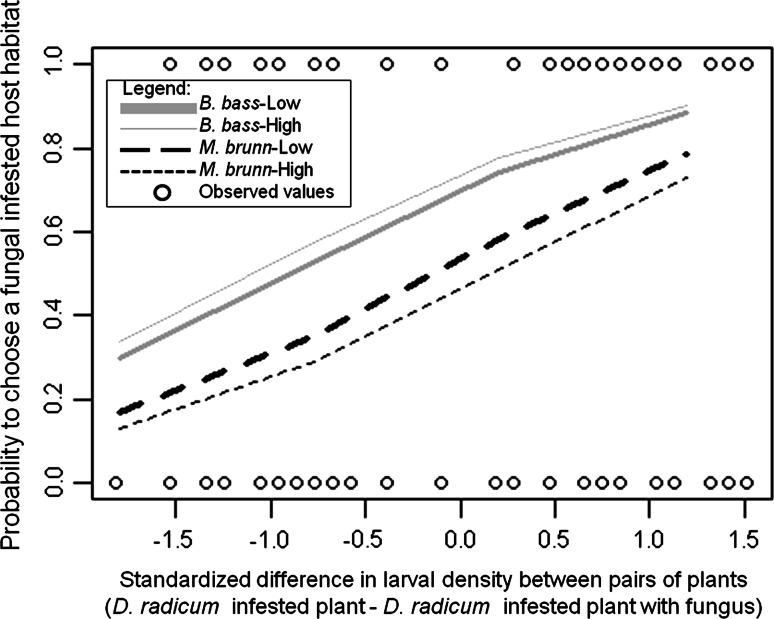
Parasitoid choice of host habitat in relation to host density and fungal species and density. The *x*-axis shows the standardized difference in larval density between pairs of plants (*negative values* signify higher larval densities in *D. radicum*-infested plants without fungi and *positive values* indicate higher densities in *D. radicum*-infested plants with fungi). The *y*-axis shows the probability of choosing a fungal infested host habitat (proportions of 0.5 equal attractions). The* lines* represent the fitted values for the four fungal treatments obtained by a Bernoulli generalized linear model applied to model 7 and *open circles* are observed values. Parasitoids are attracted to fungal infested host habitats with high densities of *D. radicum* larvae, but the attraction is higher in the presence of *B. bassiana*–Low and *B. bassiana*–High (*grey lines*) compared with *M. brunneum*–Low and *M. brunneum*–High (*black lines*)

In addition to findings in Fig. 1b and c, where the treatment combinations are analyzed individually, the results of the Bernoulli GLM (model 7) showed that differences in density of *D. radicum* larvae between plant pairs significantly influenced parasitoid choice, independently of fungal species or fungal density (Table 2). The model results also showed that, despite the larval density effect, the attraction to HIP with fungus was significantly different depending on fungal species and fungal density. Table 2 compares the three fungal treatments: *M. brunneum*–High, *M. brunneum*–Low and *B. bassiana*–Low with reference to *B. bassiana*–High, which is absent since it is the baseline factor. In the presence of *M. brunneum*–High, the probability of attraction towards a fungal infested habitat was significantly lower than when *T. rapae* encountered *B. bassiana*–High. This effect was less significant in the presence of *M. brunneum*–Low and non-significant in the presence of *B. bassiana*–Low. Figure 2 provides a visual interpretation of model 7. The results of the models 2–5 with all the individual factors (Online Resource 3) showed that, in addition to difference in larval density, fungal species or fungal density, only arm orientation had a significant effect on determining the parasitoid´s choice, when it was analyzed individually.

**Table 2:** Results of the Bernoulli generalized linear model for avoidance (*0*) or attraction (*1*) of *T. rapae* parasitoids towards highly *D. radicum*-infested plants with fungus, for a model including difference in larval density, fungal species and fungal densities

	Estimate	SE	95 % CI	99 % CI	99.5 % CI	Significance
Intercept	1.08	0.33	0.451, 1.733	0.317, 1.906	0.228, 2.030	***
Difference in larval density	0.99	0.17	0.666, 1.316	0.607, 1.373	0.572, 1.400	***
*M. brunneum*–High	−1.24	0.44	−2.118, −0.379	−2.283, −0.175	−2.385, −0.063	**
*M. brunneum*–Low	−0.93	0.45	−1.803, −0.069	−1.990, 0.091	−2.146, 0.253	*
*B. bassiana*–Low	−0.20	0.45	−1.129, 0.664	−1.294, 0.846	−1.404, 0.963	NS

*M. brunneum–High* Plants inoculated with 1 × 10^8^, * M. brunneum–Low* plants inoculated with 5 × 10^7^ conidia of *M. brunneum* mL^−1^, *Beauveria bassiana–Low* plants inoculated with 1 × 10^8^ conidia of *B. bassiana* mL^−1^, *CI* confidence interval

*NS* 95 % CI spans zero, * 95 % CI does not span zero, ** 99 % CI does not span zero, *** 99.5 % CI does not span zero

### Plant volatiles

Analysis of the volatiles trapped from HIP and HIP with fungus using multivariate statistics (after removing compounds found in UP, empty bags and soil) revealed 14 different volatile compounds (Table 3). From these compounds (COMP1–14), we only included COMP2, COMP5–6 and COMP9–14 in the PCA analysis, after discharging highly correlated compounds. Although PCA can deal with correlation (Zuur et al. 2007), those compounds which presumably measured the same underlying aspect of volatile emissions from the habitats were removed, assuming that including the redundant variables can cause the PCA to overemphasize their contribution.

**Table 3:** Relative amounts (mean ± SD) of the 14 volatile compounds (*COMP*) emitted from HIP and HIP with different fungal species (*M. brunneum* and *B. bassiana*) and densities (low and high)

Number	Compound	Retention time (min)	Kovats index (DB-WAX)	CAS number	HIP (*n* = 8)	*B. bassiana*–High (*n* = 5)	*M. brunneum*–High (*n* = 5)	*M. brunneum*–Low (*n* = 5)	*χ* ^2^ * df* = 3	*P*
ISTD1	Heptyl acetate^a^	10.75	1369	112-06-1						
ISTD2	Undecyl acetate^a^	17.93	1780	1731-81-3						
COMP1	n-butyl ether	3.59	987	142-96-1	0.89 ± 0.74	0.87 ± 0.66	0.65 ± 0.13	0.33 ± 0.45	3.531	n.s.
COMP2	Dimethyl disulfide^a^	4.87	1076	624-92-0	0.03 ± 0.06	0.04 ± 0.07	0.00 ± 0.00	0.02 ± 0.05	2.435	n.s.
COMP3	Butyl acetate	5.01	1073	123-86-4	0.21 ± 0.19	0.11 ± 0.07	0.06 ± 0.03	0.02 ± 0.04	5.560	n.s.
COMP4	Butyl propionate	6.25	1140	590-01-2	0.51 ± 0.46	0.25 ± 0.17	0.15 ± 0.05	0.04 ± 0.06	6.255	n.s.
COMP5	β-Myrcene^a^	6.69	1161	123-35-3	0.07 ± 0.08	0.08 ± 0.02	0.06 ± 0.04	0.04 ± 0.06	1.363	n.s.
COMP6	o-Xylene^a^	6.84	1178	95-47-6	1.07 ± 0.08	0.88 ± 0.41	0.87 ± 0.45	1.04 ± 0.06	0.302	n.s.
COMP7	3-Hexanol^a^	7.33	1196	623-37-0	0.00 ± 0.00	0.05 ± 0.12	0.00 ± 0.00	0.00 ± 0.00	3.600	n.s.
COMP8	Butyl butanoate	7.56	1218	109-21-7	0.13 ± 0.13	0.05 ± 0.05	0.04 ± 0.02	0.00 ± 0.00	6.815	n.s.
COMP9	1-ethyl-3-methylbenzene	8.78	1270	620-14-4	0.03 ± 0.03 a	0.03 ± 0.01 a	0.00 ± 0.01 a	0.00 ± 0.00 b	9.946	0.019
COMP10	(Z)-3-hexen-1-yl-acetate^a^	9.57	1329	3681-71-8	0.05 ± 0.07	0.00 ± 0.01	0.01 ± 0.02	0.14 ± 0.13	7.485	n.s.
COMP11	Allyl isothiocyanate	10.20	1342	57-06-7	0.02 ± 0.05	0.01 ± 0.02	0.00 ± 0.00	0.08 ± 0.08	5.746	n.s.
COMP12	Unknown	10.542	1346	_	0.05 ± 0.04 a	0.02 ± 0.01 a	0.02 ± 0.01 a	0.04 ± 0.01 a	9.725	0.021
COMP13	2-Ethylhexyl acetate	10.96	1382	103-09-3	0.08 ± 0.08	0.03 ± 0.03	0.01 ± 0.02	0.00 ± 0.01	4.532	n.s.
COMP14	1,3-Di-tert-butylbenzene^a^	11.78	1423	1014-60-4	0.00 ± 0.00 b	0.02 ± 0.02 a	0.04 ± 0.03 a	0.00 ± 0.00 b	21.484	0.002

*Different letters* indicate significant difference of pairwise comparisons of the mean relative amounts emitted from the four treatments

*CAS* Chemical Abstracts Service, *n.s.* Non-significance as indicated by Kruskal–Wallis test (*P* < 0.05)

^a^Compounds verified by comparison of retention time and mass spectra with standards

**Fig. 3:**
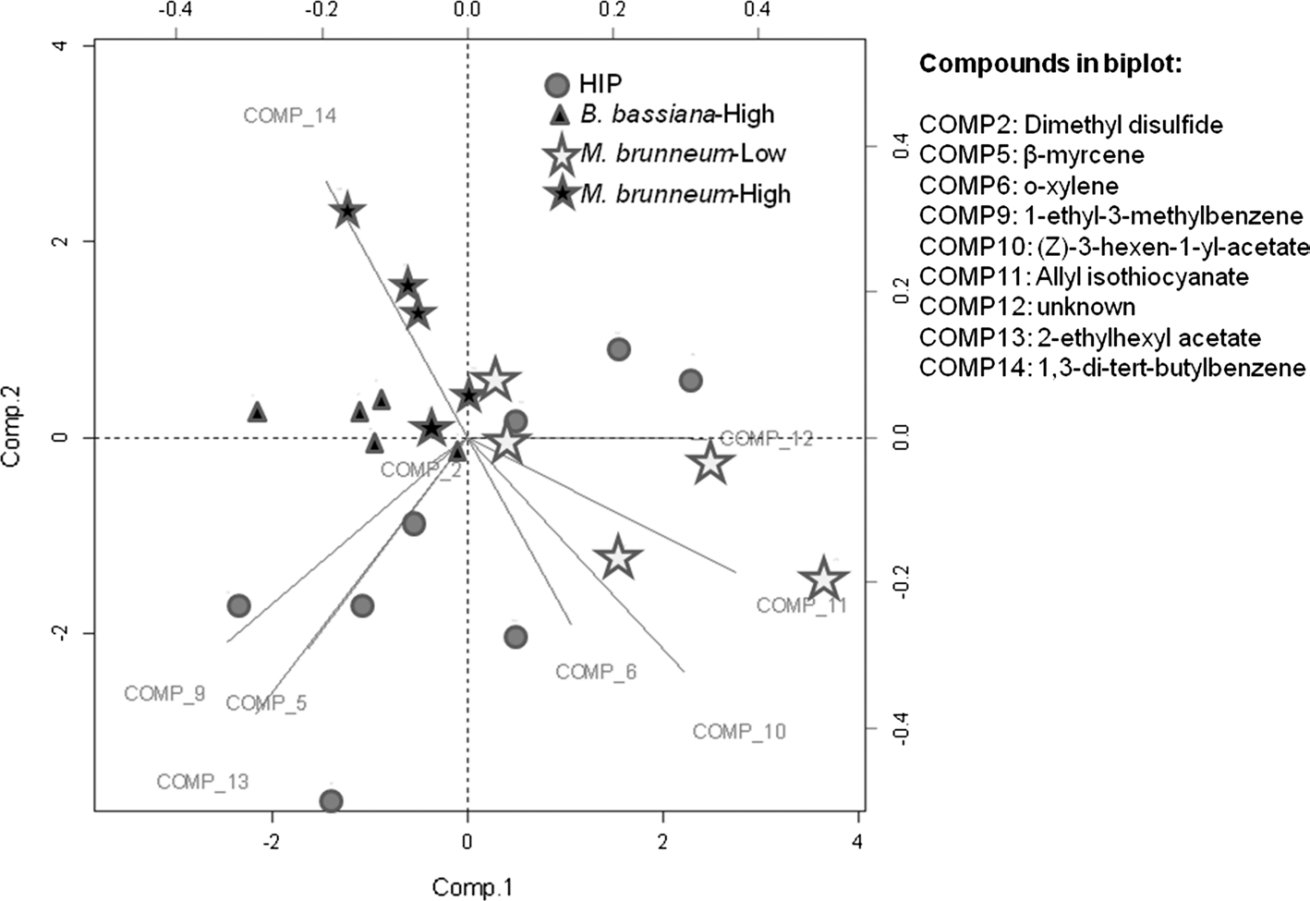
Principal component (PC) analysis biplot for nine volatile compounds identified by gas chromatography–mass spectrometry (*lines*) and the 23 plant samples. Represented treatments are: HIP (*circles*); plants heavily infested with a high density of *B. bassiana* (*B. bassiana*-*High*; *triangles*); plants heavily infested with a low density of *M. brunneum* (*M. brunneum*-*Low*; *grey stars*); and plants heavily infested with a high density of *M. brunneum* (*M. brunneum*-*High*; *black stars*). The PCs are PC1 and PC2

The first three PCs accounted for 62.40 % of the total variation of the data set. The biplot represents PC1 and PC2 (Fig. 3) and loadings of the first three PCA are presented in Online Resource 4. PC1 and PC2 were chosen because they clearly discriminated amongst the plant groups. As can be observed in Fig. 3, there are grouping clusters of samples. HIP and HIP with *M. brunneum*–Low can be differentiated from HIP *M. brunneum*–High and from *B. bassiana*–High. In the biplot the distance between the points shows the similarity in the observations (plants) based on the volatile compounds (lines). The projection of a point on a line represents the contribution of a certain compound to the volatile profile of that specific plant. Thus, the PCA loading table (Online Resource 4) indicates that 1,3-di-tert-butylbenzene was highly related to plants with *M. brunneum*–High while compounds such as dimethyl disulfide and 1-ethyl-3-methylbenzene are more representative of HIP. Finally, allyl isothiocyanate is located next to HIP with *M. brunneum*–Low.

The PerMANOVA showed that fungal species and fungal density affected the odor profile exhibited by HIP (PerMANOVA, *F*_3,22_ = 2.308, *P* < 0.001). Furthermore, Kruskal–Wallis tests followed by post hoc tests comparing the relative amounts of individual compounds emitted from HIP plants under the different treatments indicated significant differences for the compounds 1-ethyl-3-methylbenzene, unknown, and 1,3-di-tert-butylbenzene. However, only the latter compound differentiated high fungal densities of both fungal species from HIP and the plants with *M. brunneum*-Low (Table 3). This, combined with the separation in the PCA biplot, indicated differences between treatments.

## Discussion

In this study we demonstrated that the belowground foraging parasitoid *T. rapae* was less attracted to host habitats infested by entomopathogenic fungi when searching for hosts. The fungi represented an IGP risk and we found that the lower attraction depended on fungal species and density. In addition, an earlier study showed that *T. rapae* females oviposit more in healthy hosts than in hosts infected by *M. brunneum* in a dual-choice experiment (Rännbäck et al. 2015). This indicates an ability of female *T. rapae* to evaluate host quality and IGP risks to their offspring. Furthermore, we found that when hosts were present in the habitat at high densities, this factor was more important for parasitoid attraction than presence of IG predators. Successful evaluation and response to cues indicating habitat quality, such as host density and presence of IGP risk, would allow *T. rapae* to select the most profitable host patch, leading to maximal reproductive success. Similarly, other studies on herbivore-natural enemy systems have reported that parasitoids make foraging decisions based on the risk of IGP (Raymond et al. 2000; Snyder and Ives 2008; Meisner et al. 2011; Velasco-Hernández et al. 2013), which may increase parasitoid fitness.

Spatial patchiness, described as heterogeneous environments in which prey and predators are spatially distributed (Hassell and May 1988), can influence the dynamics of host-parasitoid interactions, among other factors (Hassell 2000). Locating suitable hosts is critical for parasitoid fitness and responses to cues related to high host densities should be selected for. The distribution of *T. rapae* represents an example of parasitism with a direct density-dependent pattern. Under natural conditions, parasitoids with this type of functional response are aggregated in patches of high host density (Hassell and May 1973; Jones and Hassell 1988). Indeed, high host densities were most attractive to *T. rapae* females in the present study, which is in agreement with previous findings (Neveu et al. 2002; Hemachandra et al. 2007). Similarly, entomopathogenic fungi often display clumped distributions in the soil environment (Meyling and Eilenberg 2007). In such situations, the IGP risk in the particular patch may become important during host searches, as females could disperse from patches where IGP risks are present into alternative unexploited host patches. This decision to disperse by the IG prey, *T. rapae*, when encountering entomopathogenic fungi, and the absence of the IG predator in certain patches, could lead to a higher survival rate of the IG prey. This fundamental ecological principle (dispersal–competition trade-off) has been applied to explain how spatial processes can promote coexistence between two competing species (Tilman 1994). Furthermore, besides increasing reproductive success, a preference for exploiting highly profitable host patches may also provide enemy-free space for *D. radicum* where populations are low. Both mechanisms will ultimately stabilize the host-parasitoid population dynamics (Vet 1999).

Our behavioral studies indicated that foraging *T. rapae* females reduce the IGP risk to themselves and their offspring through avoidance of host habitats with high densities of the IG predator *M. brunneum*. Avoidance responses to potential IGP risks to parasitoid offspring from entomopathogenic fungi have been observed in other studies with infected hosts (Brobyn et al. 1988; Fransen and van Lenteren 1993). However, in these studies no IGP risk was posed to the foraging parasitoid, as the fungal species tested were not pathogenic to adults. In contrast, our host-pathogen-parasitoid system included an IGP risk to both adult females and their offspring. Both fungal isolates used are pathogenic to adult *T. rapae* and to larvae of their host, *D. radicum*, with the *M. brunneum* isolate exhibiting the higher virulence (Rännbäck et al. 2015). Since the fungal species may infect the parasitoid larva inside the fungal infected host as well as the adult female parasitoid *T. rapae* during host foraging, both coincidental (IGP occurring when two species simultaneously attack a prey individual) and omnivorous (IGP occurring independently of prey consumption) IGP (Straub et al. 2008) respectively, are risks to *T. rapae*. If the foraging female fails to perceive an IGP risk and does not avoid fungal infested host habitats, coincidental IGP towards parasitoid larvae developing in infected hosts is likely. Furthermore, fungal infected *T. rapae* females can perceive an IGP risk to themselves and compensate for a reduced life expectancy through an increase in oviposition rate (Rännbäck et al. 2015).

Volatile collections from host habitats with IGP risk displayed different compound profiles, which correlated with the behavioral response observed for *T. rapae* in the bioassays.

The volatiles emitted from the plant–larvae host complex differed depending on whether the HIPs had been treated with fungus and on fungal density. The compound 1,3-di-tert-butylbenzene, an alkyl benzene, occurred only when high densities of either of the fungal isolates were present. Alkyl benzenes have previously been detected in fungal cultures (Jeleń and Wasowicz 1998). It has been reported that 1,3-di-tert-butylbenzene is released by *B. bassiana* (Crespo et al. 2008), and moreover, is emitted as the main volatile compound from mycelium cultures of another ascomycete, *Tuber borchii* Vitt (Tirillini et al. 2000). Its presence in our experimental habitats may indicate actively growing mycelium, but we cannot conclude whether 1,3-di-tert-butylbenzene originates from an interaction with cabbage roots, growth on larval cuticles, or a combination of these. Another interesting volatile is dimethyl disulfide, which we found in all treatments except *M. brunneum*–High. This compound is emitted by plants heavily infested with *D. radicum* larvae and it has been found to be attractive to the main predators of *D. radicum* in the field (Ferry et al. 2007). The absence of dimethyl disulfide in *M. brunneum*–High may have contributed to the reduced attraction of *T. rapae* to this treatment in our experiment. A substantial reduction in the feeding of *D. radicum* larvae could be evident due to the high virulence of the fungus, especially at high fungal densities (Rännbäck et al. 2015), and thus lead to lower emission rates of dimethyl disulfide. The females may use cues of host density and host quality, e.g., feeding status, fungal infection (IG predator), to detect differences between habitats and make foraging decisions. The composition and quantity of emitted volatiles constitute that cue.

Recently, entomopathogenic fungi were shown to be able to colonize the rhizosphere of several plants (Bruck 2010; Sasan and Bidochka 2012; Akutse et al. 2013; Razinger et al. 2014). The resulting mycelium could potentially be responsible for the production of volatile compounds such as 1,3-di-tert-butylbenzene, or induction of yet unknown compounds emitted by the plant. The ecological consequences of this plant-fungus interaction for other organisms are currently not fully understood. In contrast to our findings, Kepler and Bruck (2006) reported that a combination of pine tree roots and *M. brunneum* attracts larvae of the belowground herbivore *Otiorhynchus sulcatus* (F.) (Coleoptera: Curculionidae). The mechanisms behind this response are, however, unknown. Furthermore, there is increasing evidence that other root-colonizing fungi can induce changes in the profile of volatile organic compounds released by plants attacked by herbivores, resulting in the increased attraction of natural enemies (Schausberger et al. 2012; Battaglia et al. 2013; Pineda et al. 2013). However, in our system it remains to be identified which compounds are behaviorally active and whether their emission from the plant is caused by fungus, plant roots, host larvae or a combination of these.

Our findings show that host habitats infested with virulent fungi can influence the behavior of foraging parasitoids during host location. A dispersal strategy to visit patches unexploited by fungi may favor parasitoids by allowing them to forage in an enemy-free space and thus most likely achieve greater reproductive success. However, we also found that parasitoids encountering high-density host patches may ignore fungal presence in the habitat. These results emphasize the importance of understanding the behavior of foraging parasitoids when they visit patches with IG predators, since avoiding IGP risk is crucial for their survival and fitness.

### Author contribution statement

B. R., P. A., N. V. M. and L.-M. R. originally formulated the idea; B. R., P. A. and B. C. conceived and designed the experiments; L.-M. R. and N. V. M. performed insect and fungal methodologies; B. C. performed the experiments and developed the statistical models; M. B. and H. R. N. collaborated in chemical analysis; all authors wrote the manuscript.

## Electronic supplementary material

Supplementary material 1 (DOCX 14 kb)

Supplementary material 2 (PDF 71 kb)

Supplementary material 3 (DOCX 17 kb)

Supplementary material 4 (DOCX 14 kb)

## References

[CR1] Akutse KS, Maniania NK, Fiaboe KKM, Van den Berg J, Ekesi S (2013). Endophytic colonization of *Vicia faba* and *Phaseolus vulgaris* (Fabaceae) by fungal pathogens and their effects on the life-history parameters of *Liriomyza huidobrensis* (Diptera: Agromyzidae). Fungal Ecol.

[CR2] Anderson MJ (2001). A new method for non-parametric multivariate analysis of variance. Austral Ecol.

[CR3] Battaglia D (2013). Tomato below ground-above ground interactions: *Trichoderma longibrachiatum* affects the performance of *Macrosiphum euphorbiae* and its natural antagonists. Mol Plant Microbe Int.

[CR4] Baverstock J, Alderson PG, Pell JK (2005). Influence of the aphid pathogen *Pandora neoaphidis* on the foraging behaviour of the aphid parasitoid *Aphidius ervi*. Ecol Entomol.

[CR5] Baverstock Roy HE, Pell JK (2010). Entomopathogenic fungi and insect behaviour: from unsuspecting hosts to targeted vectors. Biocontrol.

[CR6] Brobyn PJ, Clark SJ, Wilding N (1988). The effect of fungus infection of *Metopolophium dirhodum* (Hom.: Aphididae) on the oviposition behaviour of the aphid parasitoid *Aphidius rhopalosiphi* (Hym.: Aphidiidae). Entomophaga.

[CR7] Brooks WM, Beckage NE, Thompson SN, Federici BA (1993). Host-parasitoid-pathogen interactions. Parasites and pathogens of insects.

[CR8] Brown PE, Anderson M (1999). Factors affecting ovipositor probing in *Trybliographa rapae*, a parasitoid of the cabbage root fly. Entomol Exp Appl.

[CR9] Bruck D (2010). Fungal entomopathogens in the rhizosphere. Biocontrol.

[CR10] Chandler D, Davidson G (2005). Evaluation of entomopathogenic fungus *Metarhizium anisopliae* against soil-dwelling stages of cabbage maggot (Diptera: Anthomyiidae) in glasshouse and field experiments and effect of fungicides on fungal activity. J Econ Entomol.

[CR11] Connor EF, Cargain MJ (1994). Density-related foraging behaviour in *Closterocerus tricinctus*, a parasitoid of the leaf-mining moth, *Cameraria hamadryadella*. Ecol Entomol.

[CR12] Crespo R, Pedrini N, Juarez MP, Dal Bello GM (2008). Volatile organic compounds released by the entomopathogenic fungus *Beauveria bassiana*. Microbiol Res.

[CR13] Dicke M, Baldwin IT (2010). The evolutionary context for herbivore-induced plant volatiles: beyond the cry for help. Trends Plant Sci.

[CR14] Dicke M, Grostal P (2001). Chemical detection of natural enemies by arthropods: an ecological perspective. Annu Rev Ecol Syst.

[CR15] Ferry A (2007). Identification of a widespread monomolecular odor differentially attractive to several *Delia radicum* ground-dwelling predators in the field. J Chem Ecol.

[CR16] Finch S (1989). Ecological considerations in the management of a *Delia* pest species in vegetable crops. Annu Rev Entomol.

[CR17] Finch S, Coaker TH (1969). A method for the continuous rearing of the cabbage root fly *Erioischia brassicae* (Bch.) and some observations on its biology. Bull Entomol Res.

[CR18] Fransen JJ, van Lenteren JC (1993). Host selection and survival of the parasitoid *Encarsia formosa* on greenhouse whitefly, *Trialeurodes vaporariorum* in the presence of hosts infected with the fungus *Aschersonia aleyrodis*. Entomol Exp Appl.

[CR19] Furlong MJ, Pell JK, Vega FE, Blackwell M (2005). Interactions between entomopathogenic fungi and arthropod natural enemies. Insect-fungal associations: ecology and evolution.

[CR20] Girling RD, Stewart-Jones A, Dherbecourt J, Staley JT, Wright DJ, Poppy GM (2011). Parasitoids select plants more heavily infested with their caterpillar hosts: a new approach to aid interpretation of plant headspace volatiles. Proc R Soc B Biol Sci.

[CR21] Guerrieri E, Lingua G, Digilio MC, Massa N, Berta G (2004). Do interactions between plant roots and the rhizosphere affect parasitoid behaviour?. Ecol Entomol.

[CR22] Hassell M (2000). Host–parasitoid population dynamics. J Anim Ecol.

[CR23] Hassell MP, May RM (1973). Stability in insect host-parasite models. J Anim Ecol.

[CR24] Hassell MP, May RM (1988). Spatial heterogeneity and the dynamics of parasitoid-host systems. Ann Zool Fenn.

[CR25] Hemachandra KS, Kuhlmann U, Mason PG, Holliday NJ (2007). Spatial patterns of *Trybliographa rapae* parasitism of *Delia radicum* larvae in oilseed rape and cauliflower. J Appl Entomol.

[CR26] Jeleń H, Wasowicz E (1998). Volatile fungal metabolites and their relation to the spoilage of agricultural commodities. Food Rev Int.

[CR27] Jones TH, Hassell MP (1988). Patterns of parasitism by *Trybliographa rapae,* a cynipid parasitoid of the cabbage root fly, under laboratory and field conditions. Ecol Entomol.

[CR28] Jönsson M, Lindkvist A, Anderson P (2005). Behavioural responses in three ichneumonid pollen beetle parasitoids to volatiles emitted from different phonological stages of oilseed rape. Entomol Exp Appl.

[CR29] Kepler RM, Bruck DJ (2006). Examination of the interaction between the black vine weevil (Coleoptera: Curculionidae) and an entomopathogenic fungus reveals a new tritrophic interaction. Environ Entomol.

[CR30] Lord JC (2001). Response of the wasp *Cephalonomia tarsalis* (Hymenoptera: Bethylidae) to *Beauveria bassiana* (Hyphomycetes: Moniliales) as free conidia or infection in its host, the sawtoothed grain beetle, *Oryzaephilus surinamensis* (Coleoptera: Silvanidae). Biol Control.

[CR31] Mburu DM, Maniania NK, Hassanali A (2013). Comparison of volatile blends and nucleotide sequences of two *Beauveria bassiana* isolates of different virulence and repellency towards the termite *Macrotermes Michealseni*. J Chem Ecol.

[CR32] Meisner M, Harmon JP, Harvey CT, Ives AR (2011). Intraguild predation on the parasitoid *Aphidius ervi* by the generalist predator *Harmonia axyridis*: the threat and its avoidance. Entomol Exp Appl.

[CR33] Meyling NV, Eilenberg J (2007). Ecology of the entomopathogenic fungi *Beauveria bassiana* and *Metarhizium anisopliae* in temperate agroecosystems: potential for conservation biological control. Biol Control.

[CR34] Meyling NV, Pell JK (2006). Detection and avoidance of an entomopathogenic fungus by a generalist insect predator. Ecol Entomol.

[CR35] Montgomery DC, Peck EA (1992). Introduction to linear regression analysis.

[CR36] Nakashima Y, Birkett MA, Pye BJ, Pickett JA, Powell W (2004). The role of semiochemicals in the avoidance of the seven-spot ladybird, *Coccinella septempunctata,* by the aphid parasitoid, *Aphidius ervi*. J Chem Ecol.

[CR37] Neveu N, Kacem N, Nenon JP (1996). A method for rearing *Trybliographa rapae* W. on *Delia radicum* L.. Bull OILB/SROP.

[CR38] Neveu N, Grandgirard J, Nenon JP, Cortesero AM (2002). Systemic release of herbivore-induced plant volatiles by turnips infested by concealed root-feeding larvae *Delia radicum* L.. J Chem Ecol.

[CR39] Nilsson U, Eriksson A, Rämert B, Anderson P (2012). Male and female *Trybliographa rapae* (Hymenoptera: Figitidae) behavioral responses to food plant, infested host plant and combined volatiles. Arthropod-Plant Interact.

[CR40] Oksanen J (2013) Vegan: Community Ecology package R package version 2.0–10

[CR41] Ormond EL, Thomas APM, Pell JK, Freeman SN, Roy HE (2011). Avoidance of a generalist entomopathogenic fungus by the ladybird, *Coccinella septempunctata*. FEMS Microbiol Ecol.

[CR42] Pierre J-S, Green RF (2008) A Bayesian approach to optimal foraging in parasitoids. In: Behavioral ecology of insect parasitoids. Blackwell, Oxford, pp 357–383

[CR43] Pineda ANA, Soler R, Weldegergis BT, Shimwela MM, Van Loon JJA, Dicke M (2013). Non-pathogenic rhizobacteria interfere with the attraction of parasitoids to aphid-induced plant volatiles via jasmonic acid signalling. Plant Cell Environ.

[CR44] Plummer M (2008). Penalized loss functions for Bayesian model comparison. Biostatistics.

[CR45] Polis GA, Myers CA, Holt RD (1989). The ecology and evolution of intraguild predation: potential competitors that eat each other. Annu Rev Ecol Syst.

[CR46] R Core Team (2012). R: a language and environment for statistical computing.

[CR47] Rännbäck L-M, Cotes B, Anderson P, Rämert B, Meyling NV (2015). Mortality risk from entomopathogenic fungi affects oviposition behavior in the parasitoid wasp *Trybliographa rapae*. J Invertebr Pathol.

[CR48] Raymond B, Darby AC, Douglas AE (2000). Intraguild predators and the spatial distribution of a parasitoid. Oecologia.

[CR49] Razinger J (2014). Direct plantlet inoculation with soil or insect-associated fungi may control cabbage root fly maggots. J Invertebr Pathol.

[CR50] Roitberg BD, Zimmermann K, Hoffmeister TS (2010). Dynamic response to danger in a parasitoid wasp. Behav Ecol Sociobiol.

[CR51] Rosenheim JA, Kaya HK, Ehler LE, Marois JJ, Jaffee BA (1995). Intraguild predation among biological control agents: theory and evidence. Biol Control.

[CR52] Roy HE, Pell JK (2000). Interactions between entomopathogenic fungi and other natural enemies: implications for biological control. Biocontrol Sci Technol.

[CR53] Roy HE, Steinkraus DC, Eilenberg J, Hajek AE, Pell JK (2006). Bizarre interactions and endgames: entomopathogenic fungi and their arthropod hosts. Annu Rev Entomol.

[CR54] Sasan RK, Bidochka MJ (2012). The insect-pathogenic fungus *Metarhizium robertsii* (Clavicipitaceae) is also an endophyte that stimulates plant root development. Am J Bot.

[CR55] Schausberger P, Peneder S, Jürschik S, Hoffmann D (2012). Mycorrhiza changes plant volatiles to attract spider mite enemies. Funct Ecol.

[CR56] Snyder WE, Ives AR, Wajenberg E, Bernstein C, Van Alphen J (2008). Behavior influences whether intra-guild predation disrupts herbivore suppression by parasitoids. Behavioral ecology of insect parasitoids.

[CR57] Soler R, Bezemer TM, Harvey JA, Wajnberg E, Colazza S (2013). Chemical ecology of insect parasitoids in a multitrophic above- and below-ground context. Chemical ecology of insect parasitoids.

[CR58] Spiegelhalter DJ, Best NG, Carlin BP, Van Der Linde A (2002). Bayesian measures of model complexity and fit. J R Stat Soc B.

[CR59] Straub CS, Finke DL, Snyder WE (2008). Are the conservation of natural enemy biodiversity and biological control compatible goals?. Biol Control.

[CR60] Su Y-S, Yajima M (2012) R2jags: a package for running jags from R. R package version 0.03–08

[CR61] Thöming G, Norli H, Saucke H, Knudsen G (2014). Pea plant volatiles guide host location behaviour in the pea moth. Arthropod Plant Interact.

[CR62] Tilman D (1994). Competition and biodiversity in spatially structured habitats. Ecology.

[CR63] Tirillini B, Verdelli G, Paolocci F, Ciccioli P, Frattoni M (2000). The volatile organic compounds from the mycelium of *Tuber borchii* Vitt. Phytochemistry.

[CR64] Velasco-Hernández MC, Ramirez-Romero R, Cicero L, Michel-Rios C, Desneux N (2013). Intraguild predation on the whitefly parasitoid *Eretmocerus eremicus* by the generalist predator *Geocoris punctipes*: a behavioral approach. PLoS One.

[CR65] Vet LM (1999). From chemical to population ecology: infochemical use in an evolutionary context. J Chem Ecol.

[CR66] Vet LEM, Dicke M (1992). Infochemical use by natural enemies of herbivores in a tritrophic context. Annu Rev Entomol.

[CR67] Waage JK (1983). Aggregation in field parasitoid populations: foraging time allocation in a population of Diadegma (Hymenoptera, Ichneumonidae). Ecol Entomol.

[CR68] Wäschke N, Meiners T, Rostas M, Wajnberg E, Colazza S (2013). Foraging strategies of parasitoids in complex chemical environments. Chemical ecology of insect parasitoids.

[CR69] Weisser WW, Houston A, Völkl W (1994). Foraging strategies in solitary parasitoids: the trade-off between female and offspring mortality risks. Evol Ecol.

[CR70] Wishart F, Monteith E (1954). *Trybliographa rapae* (Westw.) (Hymenoptera: Cynipidae). A parasite of *Hylemya* spp. (Diptera: Anthomyiidae). Can Entomol.

[CR71] Zuur AF, Ieno EN, Smith GM (2007). Analysing ecological data.

[CR72] Zuur AF, Ieno EN, Elphick CS (2010). A protocol for data exploration to avoid common statistical problems. Methods Ecol Evol.

