# Supplementary material for: Habitat selection of a parasitoid mediated by volatiles informing on host and intraguild predator densities
Source: Oecologia. 2015 May 7;179(1):151–62. doi: 10.1007/s00442-015-3326-2 (PMC4553151; doi:10.1007/s00442-015-3326-2)
Supplement: Supplementary file 1 — Supplementary material 1 (DOCX 14 kb) [file 442_2015_3326_MOESM1_ESM.docx]

**Online Resource 1** Model comparison for each dataset from the Bernoulli Generalized Linear Model using Deviance Information Criterion (DIC). Akaike Information Criterion (AIC) and Bayesian Information Criterion (BIC) are shown for comparison.

| Model number | Model with: | AIC | BIC | DIC |
| --- | --- | --- | --- | --- |
| 1 | Only an intercept | 309.2 | 312.6 | 308.2 |
| 2 | Difference in larval density | 275.5 | 282.4 | 273.4 |
| 3 | Searching time | 311.7 | 318.5 | 309.6 |
| 4 | Pot size | 311.7 | 318.5 | 309.6 |
| 5 | Arm orientation | 305.3 | 312.2 | 303.2 |
| 6 | Fungal treatments | 311.0 | 324.7 | 307.1 |
| 7 | Difference in larval density + Fungal treatment | 273.7 | 290.8 | 269.0 |
| 8 | Difference in larval density + Arm orientation | 274.5 | 284.8 | 271.6 |
| 9 | Difference in larval density + Fungal treatment + Arm orientation | 273.0 | 293.5 | 267.5 |
| 10 | Full model (all main terms) | 278.3 | 305.6 | 270.5 |
