# Supplementary figures and images for: Habitat selection of a parasitoid mediated by volatiles informing on host and intraguild predator densities
Source: Oecologia. 2015 May 7;179(1):151–62. doi: 10.1007/s00442-015-3326-2 (PMC4553151; doi:10.1007/s00442-015-3326-2)

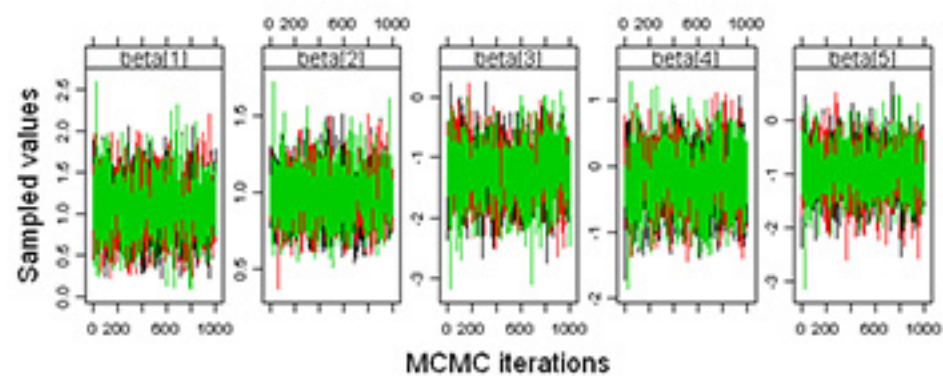

Supplement: Supplementary file 2 — Supplementary material 2 (PDF 71 kb) [file 442_2015_3326_MOESM2_ESM.pdf]
