# Supplementary material for: Habitat selection of a parasitoid mediated by volatiles informing on host and intraguild predator densities
Source: Oecologia. 2015 May 7;179(1):151–62. doi: 10.1007/s00442-015-3326-2 (PMC4553151; doi:10.1007/s00442-015-3326-2)
Supplement: Supplementary file 3 — Supplementary material 3 (DOCX 17 kb) [file 442_2015_3326_MOESM3_ESM.docx]

**Online Resource 3** Results of the Bernoulli Generalized Linear Model for avoidance (0) or attraction (1) of *Trybliographa rapae* parasitoids towards highly *Delia radicum*-infested plants (HIP) with fungus, for five models including only single factors: difference in larval density (model 2), searching time (model 3), pot size (model 4), arm orientation (model 5) and fungal treatments (model 6). *Metarhizium brunneum*-High/-Low = plants inoculated with 1 × 10^8^/5 × 10^7^ conidia of *M. brunneum* mL^-1^ ; *Beauveria bassiana*-Low = plants inoculated with 1 × 10^8^ conidia of *B. bassiana* mL^-1^. SE = standard error, CI = confidence interval. NS: 95% CI spans zero, *95% CI does not span zero, **99% CI does not span zero, ***99.5% CI does not span zero.

| Model with single factor | | Intercept  Estimate (SE) | Factor  Estimate (SE) | 99.5% CI  (Significance) |  |
| --- | --- | --- | --- | --- | --- |
| Difference in larval density | | 0.42 (0.15) | 0.88 (0.15) | 0.876, 0.891 *** | |
| Searching time | | 0.36 (0.14) | 0.10 (0.14) | 0.103, 0.104 NS | |
| Pot size | | 0.25 (0.20) | 0.19 (0.27) | 0.193, 0.198 NS | |
| Arm orientation | | 0.79 (0.22) | -0.73 (0.28) | -0.727, -0.745 ** | |
| Fungal treatments | *M. brunneum-High* | 0.72 (0.30) | -0.92 (0.39) | -0.923, -0.945* | |
|  | *M. brunneum-Low* |  | -0.11 (0.40) | -0.107, -0.111 | |
|  | *B. bassiana-Low* |  | -0.33 (0.40) | -0.331, -0.346 | |
