# Supplementary material for: Habitat selection of a parasitoid mediated by volatiles informing on host and intraguild predator densities
Source: Oecologia. 2015 May 7;179(1):151–62. doi: 10.1007/s00442-015-3326-2 (PMC4553151; doi:10.1007/s00442-015-3326-2)
Supplement: Supplementary file 4 — Supplementary material 4 (DOCX 14 kb) [file 442_2015_3326_MOESM4_ESM.docx]

**Online Resource 4** Loadings of the first three PCA axes for compounds emitted from heavily infested plants (HIP) and HIP with different fungal species (*Metarhizium brunneum* and *Beauveria bassiana*) and densities (Low and High).

| Number | Compound | PC1 | PC2 | PC3 |
| --- | --- | --- | --- | --- |
| COMP2 | Dimethyl disulfide | -0.064 | -0.044 | 0.629 |
| COMP5 | β-myrcene | -0.276 | -0.364 | -0.371 |
| COMP6 | o-xylene | 0.177 | -0.321 | -0.326 |
| COMP9 | 1-ethyl-3-methylbenzene | -0.417 | -0.353 | 0.321 |
| COMP10 | (Z)-3-hexen-1-yl-acetate | 0.373 | -0.404 | 0.118 |
| COMP11 | Allyl isothiocyanate | 0.461 | -0.232 | 0.433 |
| COMP12 | Unknown | 0.412 | -0.002 | -0.206 |
| COMP13 | 2-ethylhexyl acetate | -0.365 | -0.474 | 0.004 |
| COMP14 | 1,3-di-tert-butylbenzene | -0.245 | 0.442 | 0.115 |
